# Fabrication of Low-Temperature Sintering Building Bricks Using Drilling Cutting and Geopolymeric Technology

**DOI:** 10.3390/ma14205940

**Published:** 2021-10-10

**Authors:** Wei-Hao Lee, Yi-Che Hsieh, Hsin-Wen Wang, Yung-Chin Ding, Ta-Wui Cheng

**Affiliations:** 1 Institute of Mineral Resources Engineering, National Taipei University of Technology, Taipei 10608, Taiwan; a0925828661@gmail.com (H.-W.W.); ycding@ntut.edu.tw (Y.-C.D.); twcheng@ntut.edu.tw (T.-W.C.); 2 Department of Materials and Mineral Resources Engineering, National Taipei University of Technology, Taipei 10608, Taiwan; v2vvv222@gmail.com

**Keywords:** building brick, geopolymer, drilling cutting

## Abstract

This study explores the practicability of using drill cutting (DC) as raw material to fabricate building bricks through the high-temperature sintering method and low-temperature geopolymeric setting (LTGS) process. Drilling mud can be recycled and reutilized after certain treatment procedures and is considered as a non-hazardous waste. However, the treatment process is time-consuming and not cost-effective. For the sintering method, low porosity and high mechanical strength bricks can be sintered at temperatures above 800 °C and meet CNS standards. For the low-temperature geopolymeric setting process, sodium silicate was selected as an activating agent for geopolymerization of drill cutting. Several process parameters, such as Si_2_O/Na_2_O modulus of alkali solution and low-temperature geopolymeric setting temperature, were investigated. The physical and mechanical properties of the fabricated brick were evaluated. According to the test results, 72.4 MPa compressive strength building bricks with low porosity (13.9%) and water absorption (6.0%) can be fabricated with 2.0 Si_2_O/Na_2_O alkali solution at 500 °C. The drill cutting brick fabricated not only meets the CNS 382.R2002 common brick standard, but also solve its disposal problem.

## 1. Introduction

In oil and gas exploration drilling, drilling mud is often pumped into the drill hole to bring up the drill cutting (DC) and ensure drilling efficiency. It is estimated that about 8000 tons of DC are produced every year in Taiwan. In order to comply with environmental protection regulations, DC needs to be dewatered before landfilling. More than 3 million dollars are spent on treating this large quantity of waste [1]. Because of the limited space and short period of drilling operations, only temporary storage and treatment facilities can be managed on site, which make DC treatment very inconvenient and not cost effective. Recovered DC are commonly used to stabilize surfaces that are more vulnerable to erosion, like roads and drilling pads, or be used as aggregate and filler in concrete, brick or block manufacturing [2]. The U.S. Department of Energy has even researched the possibility of using DC as a substrate for restoring coastal wetlands [3,4,5,6]. Bernardo et al. [7] and Smith et al. [8], used drilling cutting as alternative raw material in clinker production. Abbe (2009) [9] et al. used drill cutting by melting process for tiling application.

Sintering treatment has become an alternative method to recycle and reutilize inorganic wastes and residues in the production of sintered block. Many researchers have developed various processes to fabricate brick, tile and construction products by sintering sludge, reservoir sediment, and bottom ash [10,11,12,13,14]. Since the DC is the rock debris removed from the drill hole, it mainly contains clay, quartz, and feldspar minerals. Therefore, DC can also be used as raw material for the fabrication of building bricks through the sintering process.

Besides the high temperature sintering process, geopolymerization of DC can also be an alternative to fabricate building bricks because of its SiO_2_ and Al_2_O_3_ contents [7]. These tetrahedral frameworks are linked by shared oxygens as poly(sialates) or poly(sialate–siloxo) or poly(sialate–disiloxo) depending on the SiO_2_/Al_2_O_3_ ratio in the system. The effects of Si/Al, SiO_2_/Na_2_O, and Al_2_O_3_/Na_2_O have been reported elsewhere [15,16,17,18,19,20,21]. The connection of the tetrahedral frameworks takes place via long-range covalent bonds. The dense amorphous phase geopolymer structure consists of semi-crystalline 3-D alumino-silicate microstructure [16,22,23]. However, during the condensation of tetrahedral alumini-silicate units, alkali metal ions (Na^+^ or K^+^) will be needed to balance the charge associated with the framework of tetrahedral Al [24]. Geopolymers have the advantages of a high compressive strength, fire resistance, low shrinkage, and optimal acid resistance [25,26,27].

**Figure 1:**
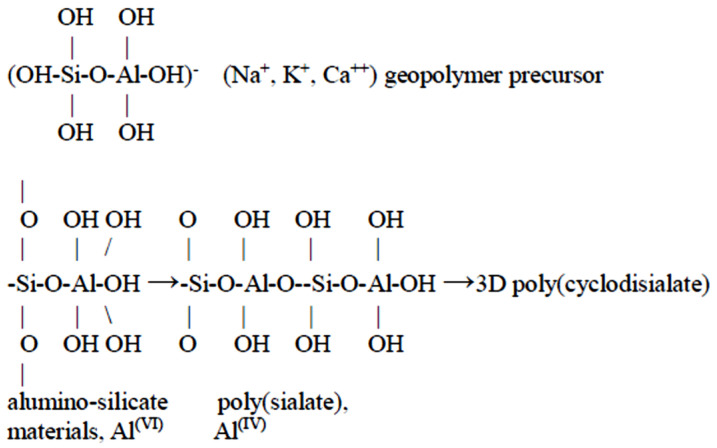
Cross-linking of LTGS geopolymeric reaction.

Low Temperature Geopolymeric Setting (LTGS) was first proposed by Joseph Davidovits [28] as a process for brick fabrication. At 450 °C geopolymeric setting temperature, LTGS brick can be fabricated with 60 MPa compressive strength. The LTGS geopolymeric cross-linking takes place with the help of a geopolymeric precursor can be expressed in Figure 1. Because DC also contains mainly soil materials, it can be a suitable raw material for the fabrication of building bricks through LTGS geopolymeric reaction.

In this study, DC was first used to fabricate building brick using the conventional process at sintering temperatures between 600 °C and 1000 °C. The physical and mechanical properties of the bricks were evaluated to determine the potential usage of DC as building brick material.

In contrast to high temperature sintering, building brick fabricated with DC through LTGS process at much lower temperature was explored. The influence of Si_2_O/Na_2_O modulus of the alkali activator and LTGS temperature on geopolymerization of DC were investigated. The mechanical and physical properties of LTGS bricks were also tested to determine the optimum process parameters. SEM analysis was used to evaluate the degree of geopolymeric reaction on the brick’s surface.

## 2. Materials and Methods

### 2.1. Materials

The DC was obtained from Chu-Huang-Keng oil drilling site located at Miao-Li County, Taiwan. In order to ensure more efficient geopolymeric reaction, it was first ground in a ball mill for 2 h for size reduction.

Alkali solutions with various SiO_2_/Na_2_O modulus were prepared by mixing sodium silicate solution (9.5 wt% Na_2_O, 29 wt% SiO_2_) and sodium hydroxide solution (NaOH) were both provided by Cheng Yi Chemical, Taiwan. A study conducted by Xu and Van Deventer [29] showed that the addition of sodium silicate solution to the sodium hydroxide solution as the alkaline activator enhanced the reaction between the source material and the solution. Tempest et al. [30], state that the sodium silicate activator rapidly dissolves to bond particles.

### 2.2. Preparation of Building Brick

The building bricks were fabricated by the conventional high temperature sintering process and LTGS processes. For the sintering process, ground DC was placed in a mold and then compacted with a steel plunger set at 140 kg/m^2^ pressure. After drying at 60 °C for 8 h and 100 °C for 24 h, bricks were sintered in a brick kiln at temperature between 600 °C and 1000 °C.

Geopolymer paste was prepared by mixing DC and alkali solution of 0, 1, 2 and 3.2 SiO_2_/Na_2_O modulus. The 0 SiO_2_/Na_2_O modulus means only NaOH was used as alkali activator. A solid/liquid ratio of 4.0 was chosen due to plastic index consideration of the paste. After thorough mixing, the paste was pressed in a brick mold at 60 kgf/cm^2^ pressure. The shaped brick was dried at room temperature for 48 h followed by oven drying at 60 °C for 8 h and 100 °C for 24 h. After drying, bricks are subjected to LTGS process at 300 °C, 400 °C, and 500 °C for 2 h. The physical and mechanical properties of the LTGS bricks were evaluated according to CNS 382.R2002 common bricks standard.

### 2.3. Materials Characterization

Compressive strength tests were carried out according to the methodology described in the standard CNS 382.R2002. As defined in CNS 9212. B7197, the constant loading rate was set as 1 mm/min.

The water absorption and bulk specific density tests, based on Archimedes’ principle, were conducted according to CNS 619.R3013.

The water absorption ($A_{W}$) was calculated as follows:

(1) $$A_{W}\left(\%\right)=\frac{W_{3}-W_{1}}{W_{3}-W_{2}}\times 100$$

The bulk specific density ($D_{b}$) was calculated as follows:

(2) $$A_{W}\left(\%\right)=\frac{W_{3}-W_{1}}{W_{3}-W_{2}}\times 100$$

where *W*1 denotes the weight of the specimen after complete drying at 105–120 °C, *W*2 represents the weight of the specimen after 24 h of soaking, and *W*3 indicates the saturation weight of the specimen.

The test results of compressive strength, flexural strength, and physical properties were all obtained after averaging the test data of five specimens.

To investigate the LTGS bricks’ microstructure, crystal phase, and chemical properties, scanning electron microscopy (SEM/EDS) analysis, X-ray diffraction (XRD) analysis, and X-ray fluorescence (XRF) were performed. We performed SEM (ZEISS Gemini SEM500) at 0.02–30 kV; XRD (Hitachi U-3310) analysis was conducted with Cu Ka, λ = 1.54060 × 10^−10^ m radiation, at 30 kV, 10 mA, and 11.4°/min; and the XRF (Hitachi X-MET8000) by Pd target to identify the LTGS bricks.

## 3. Results and Discussions

### 3.1. Conventional DC Building Brick & DC Analysis

For conventional brick fabrication, the molded DC brick were sintered at temperatures between 600 °C and 1000 °C in a brick kiln. The SEM, physical and mechanical properties of thw sintered bricks were evaluated to determine if they adhered to the CNS common brick standard.

**Figure 2:**
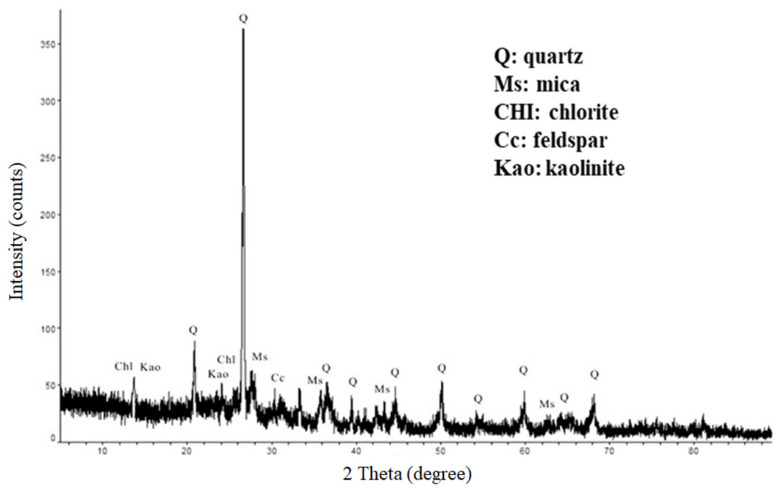
XRD spectrum of drill cutting.

The particle size of the ground particles was in the range of 0.3 μm to 250 μm, with D50 at 5.8 μm. The XRD spectrum (see Figure 2) showed quartz, kaolinite, chlorite, calcite, and mica minerals in the DC. The chemical analysis revealed that more than 81.8% of the DC was composed of SiO_2_, Al_2_O_3_, and Fe_2_O_3_, which is very similar to those found in clay minerals (see Table 1).

#### 3.1.1. SEM Microstructure Analysis

**Figure 3:**
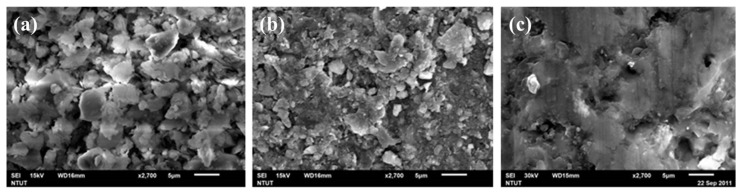
SEM images of building brick sintered at different temperature: (**a**) 600 °C; (**b**) 800 °C; (**c**) 1000 °C.

Figure 3 shows the SEM images of building bricks fabricated at 600 °C, 800 °C, and 1000 °C. The sample made at 600 °C shows a very rugged surface, and the particle boundary can be clearly identified. Therefore, it might be affected by the crystalline state of the clay and the sintering temperature, and the sample is likely to be water-absorbing to expand and then subsequently collapse. By elevating the temperature to 800 °C, a certain degree of particle sintering along with pores reduction can see on the brick surface. For brick sintered at 1000 °C temperature, most DC particles have been sintered, and the matrix gradually densifies and is form into a close-packed structure. For temperature 600 °C to 1000 °C, we can see that when the calcining temperature increases, the interface between particles shrinks, and the densification of the matrix is visible, which corresponds to the physical properties and mechanical strength test results.

#### 3.1.2. Physical Properties

The physical properties of the sintered bricks are listed in Table 2. The brick sintered at 600 °C disintegrated in boiling water during the density, porosity, and water absorption tests. Due to the very low degree sintering at 600 °C, the clay minerals of DC absorbed water, resulting in the expansion and disintegration of the brick. The bulk density of the sintered bricks slightly increased, as the temperatures increased from 700 °C to 1000 °C. According to the SEM images, the formation of pores after water escaped from the bricks at low sintering temperatures slows down as the sintering temperatures increase, which could be due to the crystal water between the layers escaping with the temperature increase, causing contact between the particles. When the particles begin to bond simultaneously, the volume of the blowhole reduces, leading the blowhole to shrink; thus, the body is gradually densified. The porosity and water adsorption decrease from 30.1% to 17.7% and 15.6% to 8.1%, respectively, as the sintering temperatures increase from 700 °C to 1000 °C. The lower water absorption of the brick sintered at a higher temperature suggests high temperature liquid phases occurred, contributing to a decrease in the pore volume, thus reducing the water absorption [31]. Similar results were also suggested by Li et al. [32] and Lin et al. [11]. The shrinkage rate of the bricks also increased from 0.5% to 5.2% due to the reduction of porosity resulting from the sintering of particles. This suggests that the high temperature sintered specimens produce a significant densification, resulting in total volume shrinkage [32]. According to the test results, it is clear that temperature plays a very important role in the physical properties of bricks fabricated through the sintering process.

#### 3.1.3. Mechanical Properties

**Figure 4:**
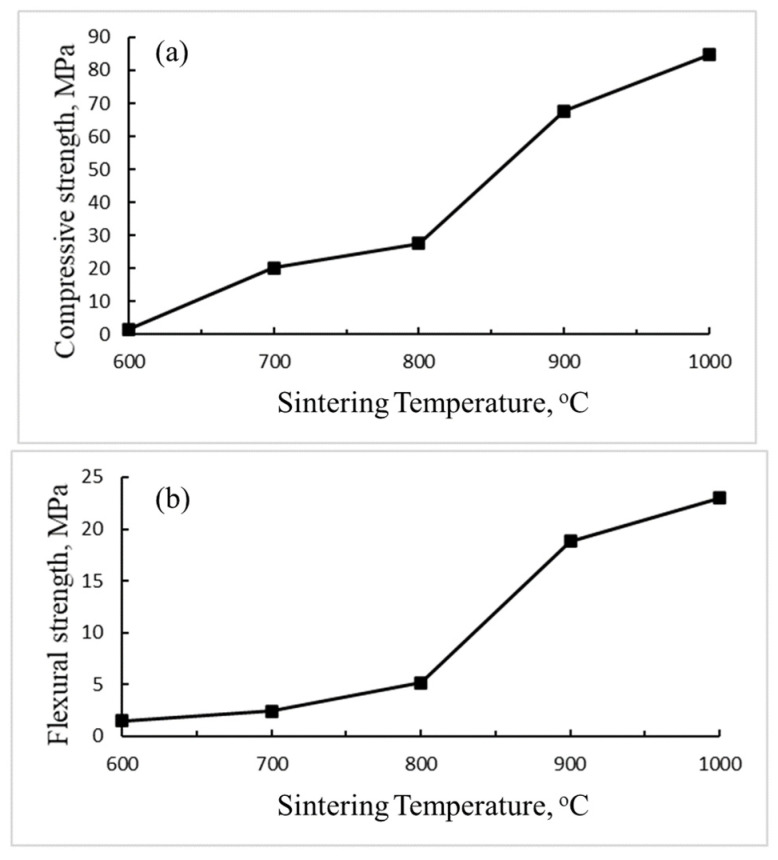
Mechanical strength of DC bricks sintered at different sintering temperatures, (**a**) compressive strength (**b**) flexural strength.

Figure 4 shows that the compressive and flexural strength of the sintered bricks increase from 2.3 MPa to 85.4 MPa and 1.6 MPa to 22.3 MPa as the sintering temperatures increase from 600 °C to 1000 °C. For brick made at 800 °C, its compressive strength (27.5 MPa) and porosity (14.6%) already meet the CNS common brick rank requirement (>15 MPa and <15%). This is because the liquid phase formed at high sintering temperature reduces open porosity, i.e., densification, and results in the higher mechanical strength of the brick [31]. These results indicate the sintering temperature is the key factor determining the quality of the bricks, and the mechanical strengths are closely related to the physical properties of the bricks [33].

### 3.2. Influence of SiO_2_/Na_2_O Modulus on the Properties of LTGS Bricks

The geopolymerization of drill cutting was done by mixing alkali solution of various SiO_2_/Na_2_O modulus with drill cutting. DC0, DC1, DC2, and DC3.2 represent LTGS bricks fabricated with 0, 1, 2, and 3.2 SiO_2_/Na_2_O modulus alkali solution at 500 °C, respectively. DC0 means only NaOH was used as the alkali solution.

#### 3.2.1. SEM Microstructure Analysis

**Figure 5:**
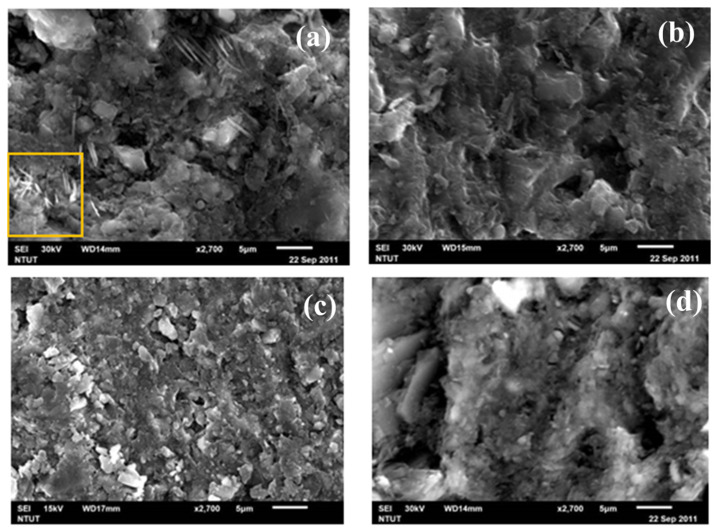
SEM images of build brick specimens made with geopolymer with various SiO_2_/Na_2_O molar ratios: (**a**) SiO_2_/Na_2_O = 0; (**b**) SiO_2_/Na_2_O = 1; (**c**) SiO_2_/Na_2_O = 2; (**d**) SiO_2_/Na_2_O = 3.7.

Figure 5 shows the SEM image of geopolymeric building bricks made with 0, 1, 2, and 3.2 SiO_2_/Na_2_O alkali solution at 500 °C. For DC0 brick, no obvious cementing can be observed and a loose, porous structure with clear particle boundary can be clearly identified. Sodium bicarbonate with needle structure is also found on DC0 specimen surface. This is believed to be the reaction of product of excess Na^+^ ions and CO_2_ in the ambient condition, as shown in Figure 5a.

For DC1 specimen, the formation of geopolymer within the particle’s interface can be observed, but with some embedded pores (Figure 5b). As the SiO_2_/Na_2_O increased to 2.0, complete geopolymerization with dense surface was formed on the DC2 specimen, and almost no particle interfaces can be seen (Figure 5c). This is probably because the ratio of Si^4+^ and Al^3+^ ions dissolved from drill cutting is well balanced to form geopolymer precursors (AlO_4_ and SiO_4_ tetrahedral unit) for the following geopolymerization [34]. For the DC3.2 specimen, the degree of geopolymerizaation reduced again and a more porous surface appeared (Figure 5d). This can be attributed to the high SiO_2_/Na_2_O modulus, i.e., low alkalinity, of the solution that reduce the dissolution of Al and Si ions, and thus, result in poorer geopolymeric reaction [35].

#### 3.2.2. Physical Properties

**Figure 6:**
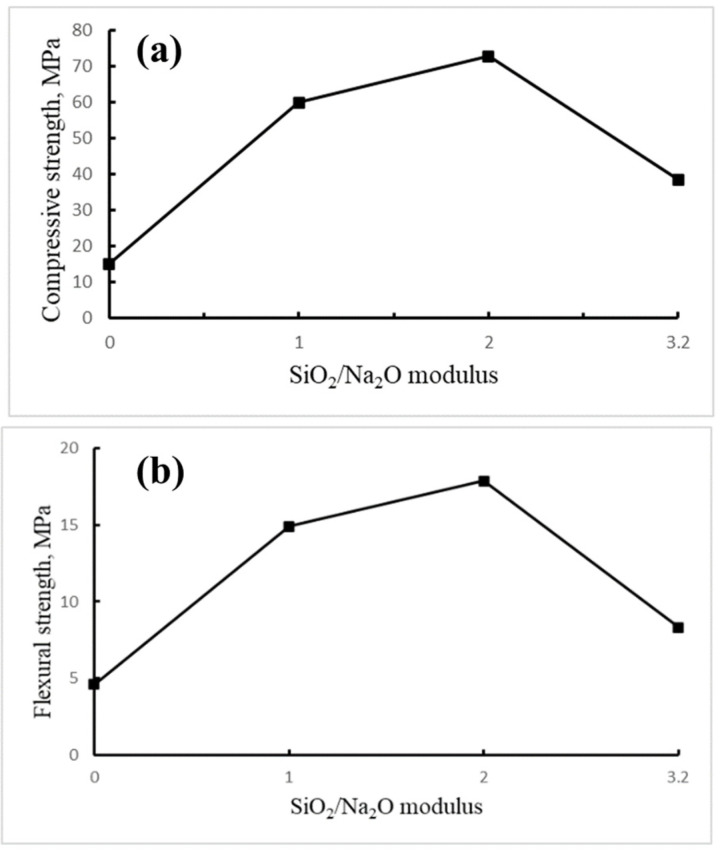
Influence of SiO_2_/Na_2_O modulus on the mechanical strength of LTGS bricks, (**a**) compressive strength (**b**) flexural strength.

Archimedes’ method was used to measure the physical properties of the LTGS bricks fabricated with various SiO_2_/Na_2_O modulus alkali solutions, as shown in Table 3. The bulk density of all bricks tested is in the range of 1.9 to 2.3 and the apparent specific gravity (apparent sp. gr.) is between 2.6 and 2.8. It is obvious that the higher the density of the bricks (DC1 and DC2), the lower the porosity and water absorption. DC2 has the lowest porosity and water adsorption due to denser surface form during geopolymerization. According to the SEM images (Figure 6), the porosity and water adsorption of the bricks are in accordance with the degree of geopolymerization, as shown in Figure 6.

#### 3.2.3. Mechanical Properties

The compressive strength of the LTGS bricks made with various SiO_2_/Na_2_O alkali solutions are shown in Figure 6. As the SiO_2_/Na_2_O modulus of the alkali solution increases from 0.0 to 2.0, the compressive strength of the brick increases from 15.5 MPa to 72.4 MPa. Normally, the low SiO_2_/Na_2_O modulus solution (high alkalinity) may accelerate the dissolution rate of the Si and Al, which are essential for the initiation of the formation of geopolymeric precursors and strength development. Gao K et al. indicated that the excess of the OH^−^ concentration may result in the early precipitation of aluminosilicate gel, causing a lower compressive strength and polycondensation [36]. This is probably the reason that DC0 and DC1 bricks have much lower mechanical strength than that of DC2 bricks. As the modulus increases to 3.2 (DC3.2), the compressive strength of the brick reduces to 39.0 MPa. This result can be attributed to the low alkalinity of the alkali solution that results in a high porosity geopolymer [35]. The flexural strength test results demonstrate the same trend as the compressive strength results. DC2 was also found to have the highest flexural strength (17.9 MPa) within all bricks fabricated.

### 3.3. Effect of Temperature on the Properties of LTGS Bricks

The 2.0 SiO_2_/Na_2_O alkali solution was used to prepare the LTGS brick because of its good physical and mechanical properties. The effect of LTGS temperature (100 °C to 500 °C) on the quality of the brick were determined by testing their physical and mechanical properties. DC100 to DC500 represent LTGS bricks fabricated at temperatures 100 °C to 500 °C

#### 3.3.1. SEM Microstructure Analysis

**Figure 7:**
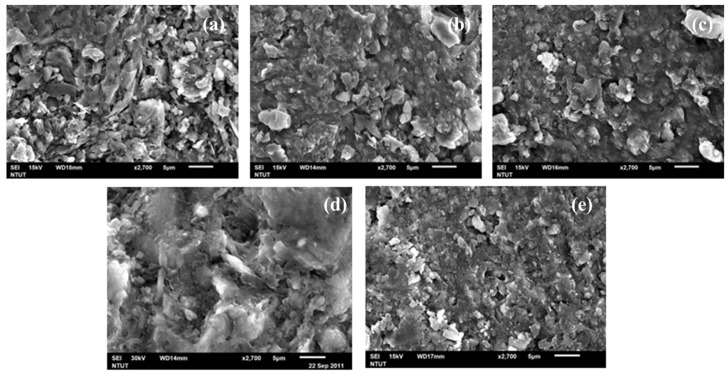
SEM images of LTGS bricks made at temperatures between 100 °C to 500 °C: (**a**)100 °C; (**b**) 200 °C; (**c**) 300 °C; (**d**) 400 °C; (**e**) 500 °C.

Figure 7 shows the SEM images of LTGS bricks fabricated at 100 °C to 500 °C. For the DC100 brick, its surface shows no obvious cementing and the drill cutting particles can be clearly identified. As the temperature increases to 200 °C, a loose porous structure with very small portion of geopolymer can be observed between particles For the DC300 brick, less dense surface with interspersed remnants of DC particles can be observed. For the LTGS brick prepared at 500 °C, complete geopolymerization of the specimen is achieved, resulting in a dense, low porous, rigid surface. This is because the higher setting temperature can lead to a better geopolymeric cross-linking structure [28]. According to the SEM analysis, the heating temperature plays a very important role in the geopolymeric reaction of the drill cutting and densification of the LTGS bricks.

#### 3.3.2. Physical Properties

The physical properties of LTGS bricks are shown in Table 3. DC100 and DC200 bricks disintegrated during the boiling step due to their low cementing and loose structure. Similar results were also reported by Mbumbia et al. (2000). [37] The density and apparent sp.gr. are in the range of 2.2 g/cm^3^–2.3 g/cm^3^ and 2.7–2.8, respectively, for LTGS bricks prepared at 300 °C and above. When comparing the porosity and water absorption of LTGS bricks, the higher the heating temperature, the lower the porosity and water absorption of the brick that were measured. This is due to the stronger geopolymeric cross-linking structure formed at a higher temperature. The porosity and water absorption of DC400 and DC500 are very similar and much lower than DC300. This suggests that bricks fabricated at temperatures as low as 400 °C with good physical properties can be achieved.

#### 3.3.3. Mechanical Properties

**Figure 8:**
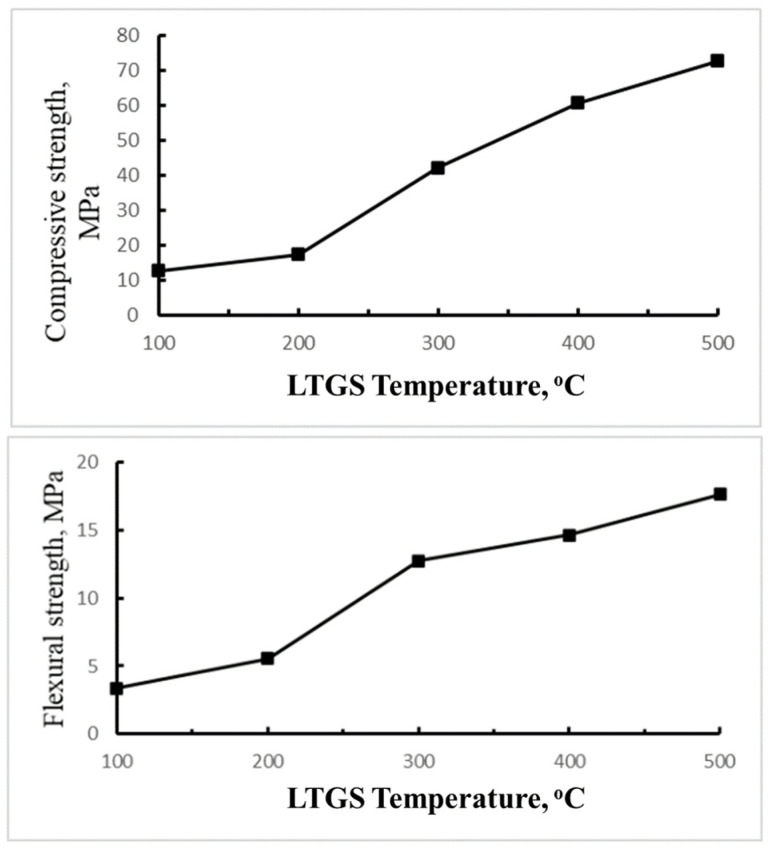
Effect of temperature on the mechanical strength of LTGS bricks, (**a**) compressive strength (**b**) flexural strength.

Figure 8 shows the compressive and flexural strength of the bricks fabricated at temperatures from 100 °C to 500 °C. The test results are in accordance with physical properties of the tested bricks, i.e., the higher the temperature, the higher the compressive and flexural strength of the bricks. As the temperature increases from 300 °C to 500 °C, bricks’ compressive and flexural strength also increases from 42.3 MPa to 72.8 MPa and 12.8 MPa to 17.7 MPa, respectively. These test results are consistent with the SEM images and physical properties of the bricks, i.e., dense surface and low porosity, as shown in Figure 8 and Table 4. It also suggests that strong geopolymeric cross-linking structure can formed at higher temperature and results in dense, rigid, and high mechanical strength LTGS brick.

#### 3.3.4. Ranking of Sintering Bricks and LTGS Bricks

The CNS (China National Standard) common brick standard (CNS382.R2002), listed in Table 5, is based on compressive strength and water absorption. Table 6 shows that the brick sintered at 800 °C, 900 °C and 1000 °C are categorized as #1, #2 and #3, respectively. It is clear that the sintering temperature determines the ranking of the bricks. Both DC1 and DC2 bricks meet CNS rank #1 requirement. LTGS bricks made at temperatures higher than 400 °C are also categorized as CNS rank #1 building bricks. According to CNS specification, bricks fabricated with drill cutting using either high temperature sintering method or the LTGS process produce satisfactory results. However, building bricks made with the LTGS process seem to be of a higher quality and are more cost effective.

## 4. Conclusions

1. According to the chemical composition and XRD analysis, drill cutting is mainly composed of SiO_2_, Al_2_O_3_, and Fe_2_O_3_ which are very similar to natural clay minerals, and can be used as raw material for building brick fabrication. In this study, building bricks were fabricated with drill cutting and met the CNS common brick standard using either high temperature sintering or LTGS process.
2. For bricks prepared with the sintering process, the sintering temperature is the key factor affecting the physical and mechanical properties of the bricks. Low porosity and water absorption bricks can be produced at high sintering temperatures because the high temperature liquid phase can decrease in the pore volume, resulting in a denser surface. The compressive strength of the brick sintered at 1000 °C can reach 85.4 MPa.
3. For building bricks fabricated with the LTGS process, it was found that the SiO_2_/Na_2_O modulus of alkali activator and temperature are two dominant factors affecting the properties of the brick. The LTGS brick made with 2.0 SiO_2_/Na_2_O alkali solution has the lowest porosity and highest mechanical strength due to complete geopolymerization of the drill cutting. The LTGS bricks fabricated at temperatures above 400 °C exhibit excellent physical and mechanical properties because of the strong geopolymeric cross-linking structure formed at higher temperatures.
4. Building bricks fabricated with either the sintering process or LTGS process all meet CNS common brick rank #1 to rank #3 requirements. However, building bricks made with the LTGS process seem to be of a higher quality and are more cost effective than those made with the high temperature sintering process. The reutilization of drill cutting for building brick fabrication not only addresses some environmental issues but also elevate its application in a more cost effective and energy efficient manner.

## Figures and Tables

**Table 1:** Chemical composition of drill cutting (Chu-Huang-Keng drilling site, Miao-Li County).

Composition	SiO_2_	Al_2_O_3_	Fe_2_O_3_	CaO	MgO	K_2_O	Na_2_O
wt.%	47.3	20.1	14.4	3.7	2.8	1.7	1.5

**Table 2:** Physical properties of building bricks made with drill cutting.

Sintering Temperature (℃)	Bulk Density (g/cm^3^)	Apparent (sp.gr.)	Porosity (%)	Water Absorption (%)	Shrinkage Rate (%)
600	-	-	-	-	0.4
700	1.9	2.8	30.1	15.6	0.5
800	1.9	2.8	29.1	14.6	1.4
900	2.1	2.7	23.7	11.4	3.0
1000	2.2	2.7	17.7	8.1	5.2

“-“: specimen disintegrated during boiling process.

**Table 3:** Influence of SiO_2_/Na_2_O modulus on the physical properties of LTGS bricks.

LTGS Bricks	SiO_2_/Na_2_O Modulus	Bulk Density (g/cm^3^)	Apparent (sp.gr.)	Porosity (%)	Water Absorption (%)	Shrinkage Rate (%)
DC0	0	1.9	2.8	33.2	17.9	0.3
DC1	1	2.2	2.6	15.4	6.9	0.3
DC2	2	2.3	2.7	13.9	6.0	0.3
DC3.2	3.2	2.1	2.7	23.1	11.2	0.3

**Table 4:** Effect of temperature on the physical properties of LTGS bricks.

LTGS Bricks	Sintering Temperature (℃)	Bulk Density (g/cm^3^)	Apparent (sp.gr.)	Porosity (%)	Water Absorption (%)	Shrinkage Rate (%)
DC100	100	-	-	-	-	0
DC200	200	-	-	-	-	0.1
DC300	300	2.2	2.8	22.4	10.5	0.2
DC400	400	2.3	2.7	15.0	6.6	0.3
DC500	5000	2.3	2.7	13.9	6.0	0.3

“-“: specimen disintegrated during boiling process.

**Table 5:** CNS common brick specifications.

Specifications	Rank #1	Rank #2	Rank #3
Water absorption (%)	<10.0	<13.0	<15.0
Compressive strength (MPa)	>30.0	>20.0	>15.0

**Table 6:** Ranking of building bricks made with high temperature sintering method and LTGS process.

Sintering Bricks	CNS Rank	LTGS Bricks	CNS Rank	LTGS Bricks	CNS Rank
800 °C	3	DC1	1	DC300	2
900 °C	2	DC2	1	DC400	1
1000 °C	1	DC3.2	2	DC500	1

## Data Availability

Data sharing is not applicable to this article.
